# Analysis of correlations between gut microbiota, stool short chain fatty acids, calprotectin and cardiometabolic risk factors in postmenopausal women with obesity: a cross-sectional study

**DOI:** 10.1186/s12967-022-03801-0

**Published:** 2022-12-12

**Authors:** Igor Łoniewski, Monika Szulińska, Mariusz Kaczmarczyk, Konrad Podsiadło, Daniel Styburski, Karolina Skonieczna-Żydecka, Paweł Bogdański

**Affiliations:** 1 Department of Biochemical Sciences, Pomeranian Medical University in Szczecin, Broniewskiego 24, 71-460 Szczecin, Poland; 2 Department of Human Nutrition and Metabolomics, Broniewskiego 24, 71-460 Szczecin, Poland; 3 Sanprobi Sp. Z O. O. Sp. K., Kurza Stopka 5/C, 70-535 Szczecin, Poland; 4 Department of Treatment of Obesity, Metabolic Disorders and Clinical Dietetics, University of Medical Sciences in Poznań, Szamarzewskiego Str. 84, 60-569 Poznań, Poland; 5 Department of Clinical Biochemistry, Pomeranian Medical University in Szczecin, Powstańców Wielkopolskich 72, 70-111 Szczecin, Poland

**Keywords:** Microbiota, SCFA, Metabolism, Cardiometabolic risk, Obesity, Menopause

## Abstract

**Background:**

Microbiota and its metabolites are known to regulate host metabolism. In cross-sectional study conducted in postmenopausal women we aimed to assess whether the microbiota, its metabolites and gut barrier integrity marker are correlated with cardiometabolic risk factors and if microbiota is different between obese and non-obese subjects.

**Methods:**

We analysed the faecal microbiota of 56 obese, postmenopausal women by means of 16S rRNA analysis. Stool short chain fatty acids, calprotectin and anthropometric, physiological and biochemical parameters were correlates to microbiome analyses.

**Results:**

Alpha-diversity was inversely correlated with lipopolysaccharide (Rho = − 0.43, FDR P (Q) = 0.004). Bray–Curtis distance based RDA revealed that visceral fat and waist circumference had a significant impact on metabolic potential (P = 0.003). Plasma glucose was positively correlated with the Coriobacteriaceae (Rho = 0.48, Q = 0.004) and its higher taxonomic ranks, up to phylum (Actinobacteria, Rho = 0.46, Q = 0.004). At the metabolic level, the strongest correlation was observed for the visceral fat (Q < 0.15), especially with the DENOVOPURINE2-PWY, PWY-841 and PWY0-162 pathways. Bacterial abundance was correlated with SCFAs, thus some microbiota-glucose relationships may be mediated by propionate, as indicated by the significant average causal mediation effect (ACME): Lachnospiraceae (ACME 1.25, 95%CI (0.10, 2.97), Firmicutes (ACME 1.28, 95%CI (0.23, 3.83)) and Tenericutes (ACME − 0.39, 95%CI (− 0.87, − 0.03)). There were significant differences in the distribution of phyla between this study and Qiita database (P < 0.0001).

**Conclusions:**

Microbiota composition and metabolic potential are associated with some CMRF and fecal SCFAs concentration in obese postmenopausal women. There is no unequivocal relationship between fecal SCFAs and the marker of intestinal barrier integrity and CMRF. Further studies with appropriately matched control groups are warranted to look for causality between SCFAs and CMRF.

**Supplementary Information:**

The online version contains supplementary material available at 10.1186/s12967-022-03801-0.

## Background

Cardiometabolic risk factors (CMRFs), including obesity, abnormal lipid profile, hypertension, insulin resistance, and aberrant glycemic control, play a role in the pathogenesis of cardiovascular diseases (CVD), which is one of the leading causes of mortality. Gut microbiota has been recently acknowledged as pivotal for human metabolic health [1, 2]. Indeed, high richness has been linked to a favourable metabolic profile [3, 4]. On the contrary, low bacterial gene counts with metabolic risk factors as demonstrated in a group of postmenopausal women with obesity [5]. Historically, germ-free mice colonized with microbiota transplant from the obese donors elevated their weight and body fat significantly more than in case of lean donors [6]. Also, a dozen of microbiota-originated or microbially-modified molecules has been recently acknowledged as factors contributing to metabolic outcome [7]. Interestingly, the production of these is strictly dependent on obesity status thus the microecological niche as elegantly shown in an in vitro study [8]. Importantly, some of microbiota-related markers were shown to be correlated significantly with type-2 diabetes (T2D) to a more considerable extent than anthropometric [9] and human genome-originated ones [10].

Excessive storage of lipids and diminished sensitive toward insulin have also been linked to mitochondrial dysfunction [11]. Interestingly, the condition of this organelle echoes the condition of microbiota [12]. For instance, in obese and diabetic patients subjected to bariatric surgery faster sugar control correlated with changes in Krebs cycle, ketone and short chain fatty acid metabolism [13]. To add, the oxidation of SCFAs inhibits lipolysis and synthesis improving lipid profile [14]. As certain antioxidants were found to increase the synthesis of SCFA [15], their use in prevention of cardiometabolic malfunctions is advised. Consequently, microbial indices might serve as markers for the early identification of metabolic disturbances [4].

Short Chain Fatty Acids (SCFA)—the main microbiota metabolites, represented mainly by acetate (C2), propionate (C3), and butyrate (C4) [16], are linked to multiple favourable metabolic functions [17]. For example, C2 is a significant source of energy for colonocytes [18], C3 might be a substrate for gluconeogenesis [17], and C4 might enter lipogenesis and cholesterol metabolism and, importantly, control appetite via the gut-brain axis [19]. Furthermore, SCFA positively affect cardiometabolic health, among other, via improving gut-barrier integrity [20–22], in particular elevating the expression of GAP and tight junction proteins [23] and, consequently, decreasing inflammation [24]. On the other hand, SCFA, however, can harm human metabolism. They are a source of about 5 -10% of the calories consumed daily, and their metabolites are involved in synthesizing lipids and glucose [25]. Moreover, faecal SCFA are positively associated with body weight [6, 26, 27], and in the obesity phenotype, there is a substantial upregulation of pathways related to SCFA production [28]. Additionally, de la Cuesta et al. [29] observed the association between SCFA excretion and gut dysbiosis, increased gut permeability, adiposity, and cardiometabolic risk factors.

Gut barrier dysfunction is increasingly recognized as a key factor in the pathogenesis of obesity, diabetes and metabolic disorders [30]. Calprotectin has the potential to be used as an indirect marker of gut permeability [31]. It is a 24 kDa dimer formed by the protein monomers S100A8 (10,835 Da) and S100A9 (13,242 Da) and makes up to 60% of the soluble proteins in the cytosol of human neutrophils [32, 33]. This marker is used mainly in older children and adults as a marker for inflammatory bowel diseases (IBD) [34].

The study of Brahe et al. of the Danish cohort of postmenopausal women with obesity showed that several gut bacterial species are linked to metabolic risk markers, also after adjustment for potential confounders [2]. They also observed that dietary fibre and fat could modify a negative correlation with insulin resistance biomarkers for *B. longum* and *F. prausnitzii*. However, the study by Brahe et al. was performed using a not very common technology and analytical pipeline. Metabolome and intestinal permeability parameters were also not analyzed. Moreover, the adjustment for differences in age, body fat percentage and diet caused the disappearance of many correlations. Therefore we decided to perform a similar analysis on the Polish population of obese postmenopausal women using anthropometric and biochemical metadata and assessing the cardiovascular function. In addition, we assessed the metabolic function of the bacteria by analyzing SCFA in the stool and the intestinal barrier status by employing faecal calprotectin. Thanks to the commonly used sequencing technology (Illumina) and bioinformatic pipelines, we were able to compare the observed results with data from a public database. We also used very demanding models of data adjustment. The study aimed to verify the following research hypotheses: (1) microbiota, SCFA and calprotectin are correlated with anthropometric, physiological and biochemical parameters in postmenopausal women suffering from obesity, and (2) stool microbiota composition is different between obese and non-obese postmenopausal women.

## Methods

The study took place from 27 February 2016 to 31 December 2017 and analysed the faecal microbiota in a population of obese postmenopausal women. It was conducted at the Department of Education and Treatment of Obesity and Metabolic Disorders University of Medical Sciences in Poznań, Poland. The protocol was registered at the U.S. National Institute of Health (ClinicalTrials.gov; Identifier: NCT03100162). Ethical approval was obtained from the Bioethical Committee of Poznan University of Medical Sciences (No. 871/2015) and prior written informed consent was obtained from all participants. The informed consent allowed samples to be used for future analyses. The material obtained during this study was analysed in a multidirectional manner, and the results were presented in peer-reviewed scientific publications [35–38].

### Subjects

The studied cohort has been described in detail previously [35]. A total of 110 postmenopausal obese women were invited to participate in the study. The inclusion criteria were as follows: (1) women aged 45–70 years, (2) ≥ 1 year since last menstruation, (3) body mass index (BMI) 30–45 kg/m^2^, (4) abdominal obesity-related waist circumference > 80 cm (International Diabetes Federation 2005); (5) body fat content, assessed by electrical bioimpedance at ≥ 33%; and (6) stable body weight in the month before the trial (permissible deviation ± 1 kg). The following criteria excluded participants from the study: (1) secondary form of obesity; (2) gastrointestinal diseases; (3) diabetes; (4) pharmacotherapy for hypertension or dyslipidemia in the three months before the trial; (5) history of use of any dietary supplements in the three months before the study; (6) intake of antibiotics within one month before the study; (7) clinically significant acute inflammation; (8) nicotine, alcohol, or drug abuse; (9) participation in weight management studies or use of medications known to alter food intake or bodyweight; (10) vegetarian dietary habits; (11) use of prebiotics- and probiotics-enriched products (for at least three weeks before the screening) and products with a high content of dietary fibre or intake of high quantities of fermented food (> 400 g/day); (12) hormone replacement therapy. Based on the inclusion and exclusion criteria, 29 women did not qualify for the study, 81 women diagnosed with obesity were eligible, and 71 were available for analysis (10 withdrew the informed consent or had cardiac events, diabetes mellitus or started supplement or antibiotic therapy). Microbiome analysis was carried out in 56 women for whom the next-generation sequencing of stool samples yielded at least 10,000 reads. A flowchart of this study is shown in Additional file 1: Fig. S1.

### Anthropometric and biochemical measurement

At enrollment, anthropometric parameters were evaluated, and laboratory tests were performed. All measurements were recorded after an overnight fast. The methods are described previously [35, 36] and included the following parameters: 1/anthropometric: weight (weight scale, metric stadiometer), waist circumference (tape measure), body composition (Bioscan 920–2); 2/vascular: blood pressure (sphygmomanometer—Omron Healthcare), pulse wave velocity and analysis (sphygmomanometer—Sphygmocor Px), augmentation index, aortic pressure and pulse pressure (applanation tonometry); 3/biochemical: glucose, uric acid, lipid profile (Lm Integrated Chemistry System Analyzer), Insulin (Immunoradiometry—Diasource Immunoassays S.A.), Lipopolysaccharide (LPS) (Kinetic Assay -Lonza, Walkersville), Tumor Necrosis Factor (TNF) -Α (Enzyme Immunoassay—DRG Instruments Gmbh), Interleukin (Il) -6 (Elisa—Drg Instruments Gmbh), vascular endothelial growth factor (vegf) (Elisa—R&D Systems), Thrombomodulin (ELISA/American Diagnostica Inc., Stamford), Von Willebrand Factor (ELISA/R&D Systems, Minneapolis).

### 16S rRNA sequencing

All steps of the 16S rRNA sequencing and reads processing were described in detail previously [38]. Briefly, the paired-end sequencing (2 × 300 bp) of the V1-V2 region of the 16S rRNA gene was performed on an Illumina MiSeq. Followed by an initial quality check, reads were processed using QIIME 2 [39] and Deblur denoising algorithm [40] which resulted in a construction of 4,716 sub operational taxonomic units, sOTU. The count per sample summary was the following: minimum 3,958, median 7,128, maximum 23,127 sub-operational taxonomic units, sOTUs. Refined taxonomic classification using species-dependent prior probabilities rather than uniform species distribution was accomplished with the q2-clawback plugin [41]. Representative sequences were used to predict functional profiles of the gut community by the PICRUSt2.

For validation, sequence data and metadata of the existing studies were queried and obtained from Qiita (https://www.qiitaucsd.edu) using the Redbiom tool [42]. First, female samples (human gut) that contained a specific set of features were identified: age (> 55 years), race (Caucasian), and BMI (BMI 18.5–24.99, BMI-NORMAL, n = 1,293, BMI ≥ 25, BMI-HIGH, n = 875). Then, the sequence data (150 nucleotides V4 16S processed with Deblur) with abundance information and metadata were downloaded for the identified samples. Data from the following Qiita studies (ID) were used: 10,317, 11,710, and 10,988. Taxonomic assignment was performed as described above, except that the V4 region was extracted from Greengenes reference sequences before classifier training, and classification was performed using taxonomic weights assembled for downloaded data.

### SCFA

The following SCFA were analysed: acetic acid (C2), propionic acid (C3), butyric acid (C4), valeric acid (C5) and hexanoic acid (C6). Fecal samples (~ 40 mg) were mixed with 0.5 ml, a mixture of acetonitrile and water (50% ACN: 50% H2O) and vortexed by 30 min. Samples were kept for 5 min. on the ice to complete protein precipitation. After centrifugation for 10 min at 4 °C at 5000 rpm and filtered through a syringe filter 0,22 µm samples were transferred to HPLC vials and analyzed by the present LC–MS technique.

An Sciex Triple TOF 6600 + equipped with an ExionLC AD series was used for the analysis of SCFA. The LC flow rate was 0.3 mL/min. The column used for the analysis was a Kinetex Polar 2.6 µm (50 mm × 3 mm). The column temperature and auto sampler were maintained at 20 °C and 4 °C, respectively. 1 µL of sample was used for the injection volume. Samples were analyzed using 10 mM ammonium formate (from VWR, Leuven, Belgium) in 80% methanol (LC–MS grade from VWR, Gliwice, Poland) with 20% ultrafiltrated water (mobile phase A) and acetonitrile (LC–MS grade from VWR, Fontenay-sous-Bois, France) (mobile phase B). The isocratic elution was 50% mobile phase A and 50% mobile phase B. The total run was 6 min. The Triple TOF 6600 + system was equipped with an electrospray ionization (ESI) and Atmospheric-pressure chemical ionization (APCI) source operated in positive and negative-ion detection mode. Nitrogen gas was used for nebulization, desolvation, and collision. The source parameters were: gas temperature of 150 °C, a Source gas 1 and source gas 2 pressure of 50 psi and capillary voltage of 3500 V for negative polarity. A standard curve was prepared by using serial dilutions of the standard mix of C2, C3, C4, C5 and C6 purchased from Sigma-Aldrich (Steinheim, Germany).

### Calprotectin

Concentrations of faecal calprotectin were determined by immunoenzymatic methods using commercial Enzyme-Linked Immunosorbent Assay (ELISA) tests (Immunodiagnostik). If the absorbance was outside of the standard curve, the sample was not included in subsequent analyses. As the dilution factor for calprotectin was equal to 2500 and the highest standard concentrations was 840 ng/mL—the highest concentration we were able to measure was 2100.

### Statistical analysis

Alpha diversity indices (number of observed sOTUs in the sample, Pielou’s evenness, Shannon’s diversity, Faith’s PD) and Bray–Curtis distance were calculated from the rarefied samples (to 3,777 sOTUs). Compositional abundance count data were transformed as follows. First, for each sample, 128 Monte Carlo instances were drawn from the Dirichlet distribution. Then, each instance was converted using the centered log-ratio transformation (ALDEx2 package). Analyses were conducted for each instance, and the results (P values, coefficients) were averaged over the instances.

To search for community composition patterns and their correlation with anthropometric, physiological and biochemical variables, the distance-based redundancy analysis (db-RDA) was used. The function *capscale* from the vegan package was used to perform db-RDA with a Bray–Curtis dissimilarity matrix. Selection of explanatory variables for db-RDA was done using a function *ordistep* (vegan) and forward and backward stepwise search and 999 permutations. The db-RDA ordination triplots were created on scores obtained from the *ordistep* selected models using scaling focused on correlations (scaling 2), thereby approximating the linear correlation between the bacterial features, between bacterial features and women’s characteristics, and between women’s characteristics.

To assess the relationship between variables, Spearman correlation or regression analysis was used. To infer a causal relationship between gut microbiota and parameters a mediation analysis was performed. It has been assumed that the mediational models are correct, i.e. gut microbiome features represent the causal (independent variables, IV), women’s characteristics (parameters) are the outcomes (dependent variables, DV), and the mediators—short chain fatty acids—are presumed to affect the outcomes. Mediation was established if a mediator effect on the DV was significant after controlling for the IV. A significant total effect (TE, a total effect of the IV on the DV without mediator) was assumed to be a prerequisite for establishing the causal mediation effect. The total effect is a sum of the indirect effect (ACME, average causal mediation effect) and direct effect (ADE, average direct effect). The significance and 95% confidence intervals were established using bootstrapping procedure. Mediation analysis was conducted using the mediation package [43].

To test for a difference in the abundance in the two datasets (Polish women and Qiita data), the Xdc.sevsample function in the HMP package [44] was used. The function performs a multivariate test for differences in the overall composition between groups assuming Dirichlet-multinomial distribution by testing for a difference in the mean distribution of each taxon across groups while accounting for the overdispersion in the count data.

The Benjamini–Hochberg procedure was used to control the false discovery rate (FDR).

### Power analysis

For the parameters and microbiota-related features (bacterial taxa or functional modules) correlation (N = 56), we used the pwr.r.test in the R package (considering r = 0.5 as a medium effect) and achieved a statistical power of 98% and 76% (considering multiple testing as per Bonferroni correction assuming the number of features N_features_ = 50).

## Results

**Fig. 1:**
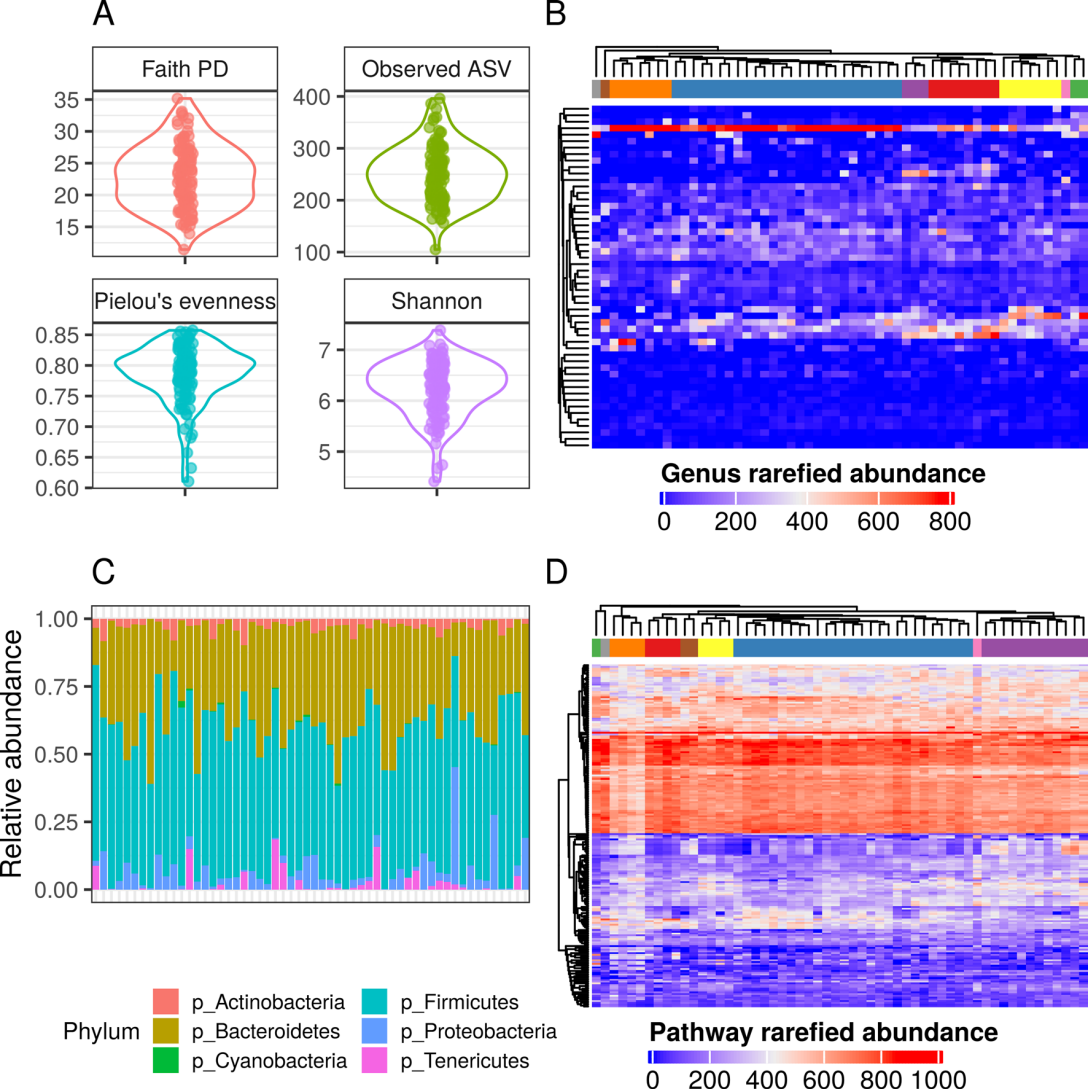
Alpha- and beta-diversity of the gut microbiota in postmenopausal women. **A** violin plots of alpha-diversity indices; **B** heatmap of the genus-level rarefied abundance, columns (samples) and rows (taxa) were subjected to average linkage method and genus-level Bray-Curtis distance hierarchical clustering, top annotation—color bars reflect identified clusters, **C** bar plot of relative abundance on phylum level, **D** heatmap of the metabolic pathway-level rarefied abundance, columns (samples) and rows (taxa) were subjected to average linkage method and genus-level Bray-Curtis distance hierarchical clustering, top annotation—color bars reflect identified clusters; Samples were grouped using hierarchical clustering based on the inter-sample genus-level (Fig. 1B) or metabolic pathway-level (Fig. 1D) Bray-Curtis dissimilarities calculated using the rarefied abundance table. **B** A high taxonomic diversity which was highlighted by the presence of 9 clusters of unequal size containing from just 1 to 26 samples. **D** two larger clusters, containing 27 and 12 samples, were identified. Bray-Curtis distances calculated on higher taxonomic levels did not affect clustering implying a persistent high taxonomic diversity in this group (Additional file 1: Fig. S2)

**Fig. 2:**
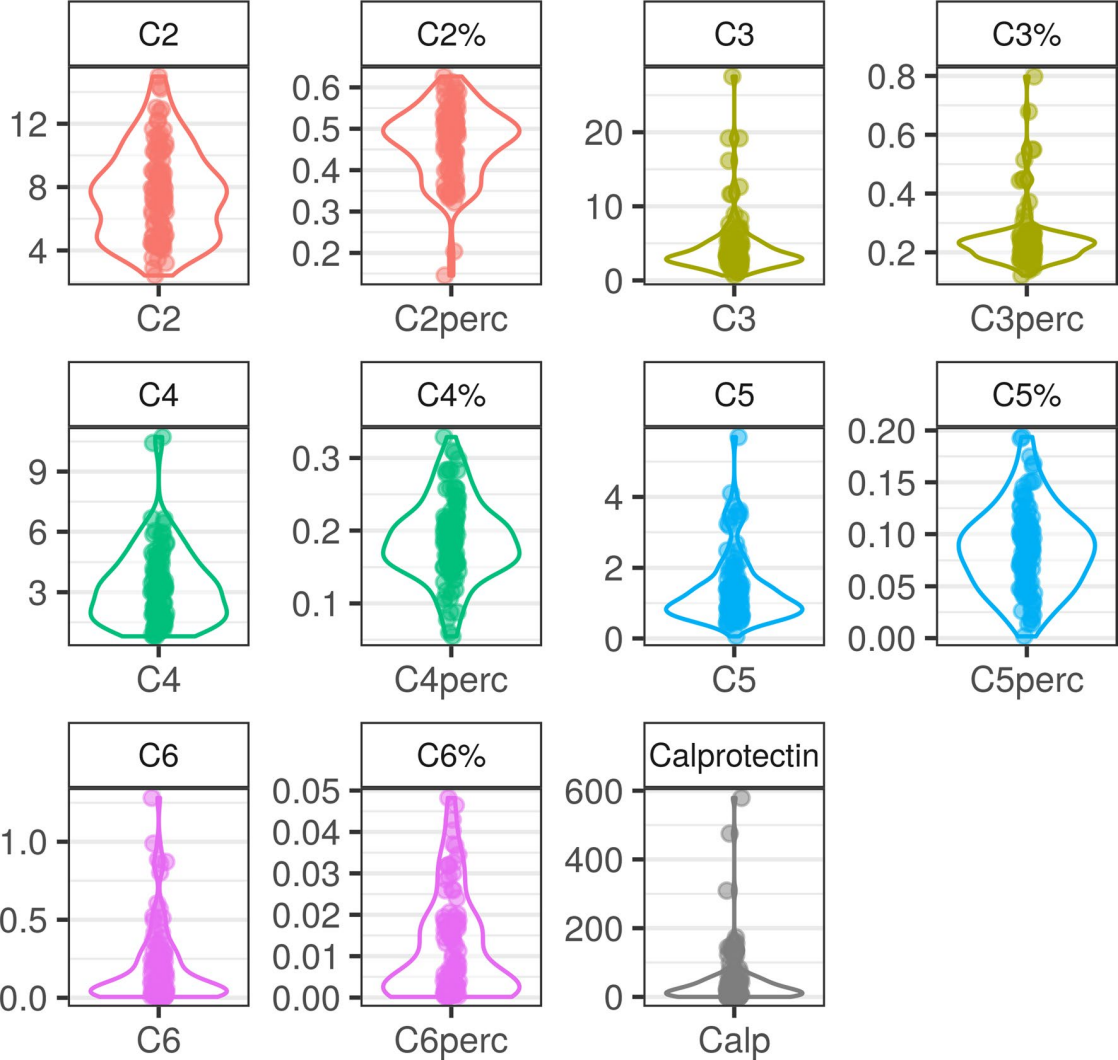
Violin plots of SCFA [nM/mg] and calprotectin concentration [ug/ml] in stool. C2—acetic acid; C2r—The ratio of acetic acid to all analyzed SCFA; C3—propionic acid, C3r—The ratio of propionic acid to all analyzed SCFA; C4—butyric acid; C4r -The ratio of butyric acid to all analyzed SCFA, C5—valeric acid; C5r—The ratio of valeric acid to all analyzed SCFA; C6—hexanoic acid; C6r—The ratio of hexanoic acid to all analyzed SCFA

The summary of demographic, clinical characteristics, and metabolic parameters are presented in Table 1. A summary of the gut microbiome data is shown in Fig. 1 and Additional file 1: Fig. S2. SCFA and calprotectin concentration were shown in Table 1 and in Fig. 2. The concentration of acetic acid (C2) in the group varied widely and ranged from 2.42 µM/ml to 15 µM/ ml. Almost in every case, the percentage of this acid was the highest among the tested acids and was most often in the range from 30 to 60% in comparison to the other tested short-chain fatty acids (SCFA). The concentration of propionic acid ranged from 0.661 µM/ml to 27 µM/ml, butyric acid from 0.7 µM/ml to 10 µM/mg, valeric acid (C5) from 0.0588 to 5.7 µM/ml and caproic acid (C6) from 0.00577 µM/ml to 1.28 µM/ml. The data distribution, IQR and median for C3, C4, C5 and C6 indicate that the data were accumulated at the lower end of the concentration ranges. The percentage of C3 acid was in the range of 12%—80%, C4 5%—33%, C5 0.1—19% and C6 0.01%—5%. The highest concentration of calprotectin in the group was 579 µg/ml however the data distribution shows that most of the results were below 100 µg/ml. Determination limit of calprotectin was 2.267 ng/ml.

**Table 1:** Descriptive statistics of selected parameters

Variable	n	Mean	SD	1st Q	Median	3rd Q
C6r	52	0.012	0.013	0.002	0.008	0.018
C6 [nM/mg]	52	0.202	0.261	0.027	0.102	0.293
C5r	52	0.085	0.041	0.057	0.089	0.113
C5 [nM/mg]	52	1.28	0.83	0.69	1.06	1.55
C4r	52	0.195	0.059	0.158	0.195	0.227
C4 [nM/mg]	52	3.18	1.69	1.82	3.02	4.46
C3r	52	0.234	0.084	0.183	0.221	0.255
C3 [nM/mg]	52	4.08	3.50	2.44	3.19	4.36
C2r	52	0.473	0.084	0.411	0.482	0.532
C2 [nM/mg]	52	7.40	2.89	4.82	7.25	9.07
Calprotectin [ug/ml]	55	64.3	107.0	13.2	28.1	56.1
TM [ng/ml]	56	4.17	0.683	3.70	4.25	4.70
vWF [IU/dL]	56	83.7	5.85	78.8	83.0	87.6
Uric Acid [mg/dL]	56	5.61	0.976	5.20	5.60	6.20
VEGF [pg/ml]	56	147	25	140	149	165
PWV [m/s]	56	7.16	1.01	6.38	7.30	7.78
PWA AP mmHg	55	13.9	7.6	9.0	12.0	18.5
PWA ALX mmHg	56	32.8	11.3	26.3	33.5	40.3
PWA SP mmHg	56	129	12	121	129	136
PWA PP mmHg	55	42.9	9.0	36.5	43.0	50.0
LPS [pg/ml]	56	1039	566	636	903	1267
IL6 [pg/ml]	56	454	56	423	446	501
TNF [ng/L]	56	1.07	0.35	0.88	0.99	1.22
CRP (log)	51	1.35	0.73	0.90	1.55	1.78
Insulin [mU/ml]	56	31.4	11.6	22.8	32.4	36.9
Glucose [mg/dL]	56	97.1	11.1	90.0	98.0	102.0
DBP mmHg	56	81.9	7.7	77.8	81.0	88.0
SBP mmHg	56	134	11	124	138	141
HR [bpm]	56	75.0	8.3	69.0	76.0	79.3
TG (log)	56	4.9	0.43	4.6	4.9	5.1
HDL (log)	56	3.9	0.2	3.8	3.9	4.1
LDL [mg/dL]	56	125	40	98	128	144
TC [mg/dL]	56	214	39	192	211	234
FFMH%	56	76.5	2.9	74.7	75.8	77.6
FFM%	56	46.5	7.7	43.1	45.9	51.9
FFM	56	44.8	6.2	40.9	44.8	47.2
Fat%	56	51.5	6.3	47.0	53.2	54.9
FM	56	47.7	10.1	40.2	47.1	52.5
TBW%	56	36.8	4.9	34.4	35.9	38.8
TBW	56	34.1	5.1	31.7	33.9	36.5
Subcutaneous fat	56	287	62	255	282	305
Visceral fat	56	218	58	174	217	239
WC [cm]	56	110	9	105	110	116
Body Mass [kg]	56	93.2	11.8	85.1	92.6	99.8
BMI [kg/m^2^]	56	35.9	4.0	32.6	35.7	38.8

C2, acetic acid; C2r, The ratio of acetic acid to all analyzed SCFAs; C3, propionic acid, C3r, The ratio of propionic acid to all analyzed SCFAs ; C4, butyric acid; C4%, The ratio of butyric acid to all analyzed SCFAs, C5, valeric acid; C5r, The ratio of valeric acid to all analyzed SCFAs; C6, hexanoic acid; C6%, The ratio of hexanoic acid to all analyzed SCFAs ; ™, thrombomodulin; vWF, von Willebrand factor; VEGF: vascular endothelial growth factor; PWV, pulse wave velocity; PWA AP, pulse wave analysis augmentation pressure; PWA Alx, pulse wave analysis augmentation index; PWA SP, pulse wave analysis systolic pressure; PWA PP, pulse wave analysis pulse pressure; LPS, lipopolysaccharide ; Il-6, interleukin-6; TNF, tumor necrosis factor alpha; CRP, C-reactive protein; DBP, diastolic blood pressure; SBP, systolic blood pressure; HR, heart rate; TG, triglycerides; HDL, high-density lipoprotein cholesterol; LDL, low-density lipoprotein cholesterol; TC, total cholesterol; FFMH, fat-free mass hydration; FFM, fat-free mass; FM, fat mass; TBW, total body water; WC, waist circumference; BMI, body mass index

### Gut microbiome in post-menopausal women with and without obesity

In total, there were 1,293 samples from Caucasian women aged over 55 and BMI ≥ 25 (BMI-HIGH) and 875 with BMI of 18.5–24.99 (BMI-NORMAL). The relative abundances of common phyla are summarized in Table 2. To test for differences between the groups, we conducted a multivariate test for differences in the overall composition. Overall, we found significant differences in the distribution of phyla between this study and BMI-HIGH (Xdc = 415.4, P < 0.0001), as well as in this study, BMI-NORMAL (Xdc = 411.3, P < 0.0001) and BMI-HIGH vs. BMI-NORMAL (Xdc = 210.6, P < 0.0001). Tests of other taxonomic levels were also conducted, and the results are shown in Additional file 1: Table S2.

**Table 2.** Phylum-level abundance in the validation data and this study

	This study (n = 56)	BMI-NORMAL (n = 1293)	BMI-HIGH (n = 875)
Actinobacteria	3.06%	1.09%	0.91%
Bacteroidetes	36.1%	27.8%	30.1%
Cyanobacteria	0.13%	0.14%	0.14%
Firmicutes	52.7%	34.2%	33.2%
Fusobacteria	0.016%	0.51%	0.23%
Proteobacteria	5.82%	32.8%	32.9%
Tenericutes	2.08%	0.80%	0.50%

BMI-NORMAL: BMI 18.5-24.99 kg/m^2^, BMI-HIGH: BMI ≥ 25 kg/m^2^

### Gut microbiota (SCFA, Calprotectin) is correlated with host-specific characteristics

**Fig. 3:**
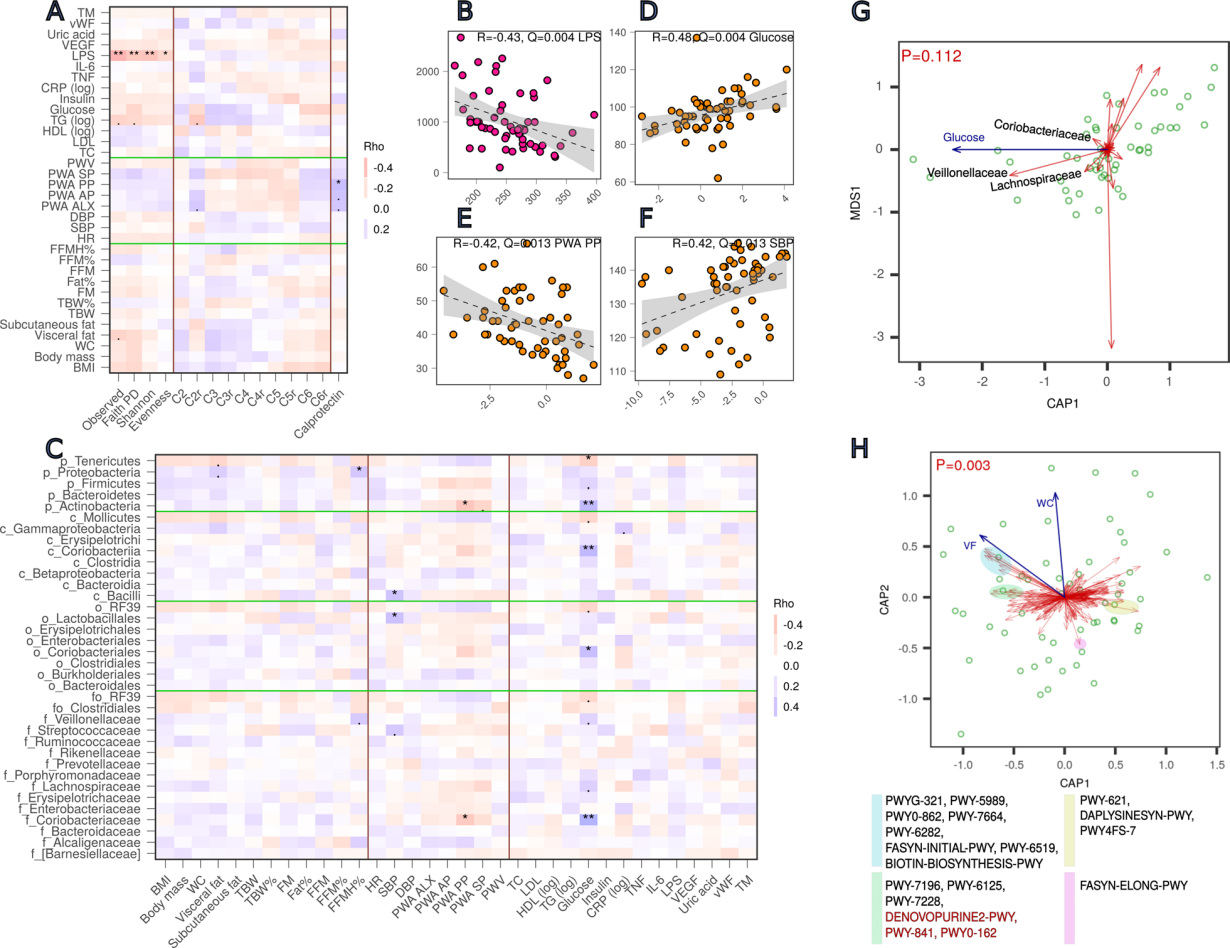
Relationship between microbiota, short chain fatty acids, calprotectin and anthropometric, physiological and biochemical parameters. **A** Spearman correlation of the alpha-diversity indices, short chain fatty acids and calprotectin (separated by vertical brown lines) with anthropometric, physiological and biochemical parameters (the three groups of parameters are separated by horizontal green lines); **C** Spearman correlation of the bacterial abundance at the family, order, class and phylum levels (separated by horizontal green lines) with anthropometric, physiological and biochemical parameters (separated by vertical brown lines); **B**, **D**, **E**, **F** pairwise scatterplots illustrating the relationship between two variables, observed number of unique features and LPS (**B**), abundance of the family Coriobacteriaceae and plasma glucose (**D**) or PWA PP (**E**), abundance of order Lactobacillales and systolic blood pressure (SBP); **G**, **H** Bray-Curtis distance-based redundancy analysis (db-RDA) ordination plots with scaling focused on correlative relationships between explanatory variables and bacterial taxa (**G**) or metabolic pathway abundance (**H**). Green points represent samples (women), red arrows represent bacteria or metabolic pathways, blue arrows represent explanatory variables. In **H**, colored areas represent the clusters of pathways that correlate with VF or WC according to the strength and direction of the relationship: Blue: stronger positive correlation with VF, green: weaker positive correlation with VF (marked red are pathways that re-appear in an univariate analysis), yellow: negative correlation with VF, pink: negative correlation with WC, VF—visceral fat, WC—waist circumference. Mapping from FDR adjusted P values ranges to symbols: 0–0.001 '***', 0.001–0.01 '**', 0.01–0.05 '*', 0.05–0.1 '.', 0.1—1.0 no symbol, Q—FDR adjusted P, Rho—Spearman correlation coefficient; C2r—ratio of acetic acid, C3r—ratio of propionic acid; C4r—ratio of butyric acid, C5r—ratio of valeric acid, C6r—ratio of hexanoic acid (to all analyzed SCFA)

All alpha-diversity indices were negatively correlated with LPS, however, the strongest relationship was found only for the observed number of unique features (sOTU, Spearman coefficient (Rho) = − 0.43, FDR P (Q) = 0.004, Fig. 3B). Two other parameters (triglycerides and visceral fat) showed a weak inverse correlation with the observed number of sOTU, yet have become insignificant after FDR correction. Calprotectin showed a weak positive correlation with PWA PP (Rho = 0.39, Q = 0.038, Fig. 3A). Short chain fatty acids were not correlated with any anthropometric, physiological and biochemical traits. Interestingly, SCAFs were correlated with alpha-diversity, C2 and C3 inversely, whereas C5 and C6 positively (Additional file 1: Fig. S3).

To assess the effect of parameters upon the gut community composition and predicted metabolic potential, a Bray–Curtis distance based Redundancy Analysis (db-RDA) was performed. The Bray–Curtis dissimilarities were calculated at all taxonomic levels (from species to phylum) as well as for predicted metabolic MetaCyc pathway abundances. Models were fitted separately for three groups of parameters, i.e. anthropometric (A model), physiological (P model) and biochemical (B model). Using the *ordistep* function from the vegan package, the simplified models were constructed in which the best (significant) explanatory variables were included. A summary of the full and simplified models with their best explanatory variables and the amount of explained variance is presented in Additional file 1: Table S1. Overall, based on the Monte Carlo permutation test, a null hypothesis of independence between the community data and constraints (explanatory variables) could be rejected for one model, i.e. MetaCyc pathways level db-RDA with anthropometric explanatory variables (visceral fat and waist circumference, Monte Carlo permutation test P = 0.003, Fig. 3H). As shown in the ordination triplot, several pathways showed a correlation with the visceral fat (VF). For example, an abundance of the PWYG-321, PWY-5989, PWY-7664, PWY0-862, PWY-6282, FASYN-INITIAL-PWY, PWY-6519, BIOTIN-BIOSYNTHESIS-PWY, PWY-6125, PWY-7196 correlated positively, whereas PWY-621 pathway was inversely correlated with the visceral fat. On the contrary, the DAPLYSINESYN-PWY showed a negative correlation with the waist circumference. However, the constrained variance in the community data (explained by these two explanatory variables) was only 9.4% (adjusted 5.9%), thus contributing a low fraction of the observed total variance in metabolic potential. Three other explanatory variables were selected as significantly explaining community variation (PWA PP at the phylum level, glucose at the genus and family level, insulin at the metabolic level), however, the global permutation tests were not significant (Fig. 3F, the family level, Monte Carlo permutation test P = 0.112). Bacteria that showed the highest correlation with plasma glucose concentration belonged to the family Coriobacteriaceae, Veillonellaceae and Lachnospiraceae.

In addition to multivariate analysis, we also performed an univariate correlation in which pairwise relationships between bacterial taxa/pathways and parameters were examined (Fig. 3C). In line with the results of multivariate methods, plasma glucose showed a positive correlation with the family Coriobacteriaceae (Rho = 0.48, Q = 0.004, Fig. 3D) and taxonomic rank to which it belongs: phylum Actinobacteria (Rho = 0.46, Q = 0.004), class Coriobacteria (Rho = 0.46, Q = 0.005), order Coriobacteriales (Rho = 0.43, Q = 0.011). Abundances of Lachnospiraceae and Veillonellaceae were also positively correlated with glucose (Q < 0.1). A similar pattern with an opposite relationship (a negative correlation), although less consistent within the taxonomic hierarchy, was observed for the PWA PP and pulse pressure (Fig. 3E). At the species level, *Bacteroides caccae* exhibited a negative relationship with waist circumference (Rho =  − 0.43, Q = 0.038). Regarding metabolic pathways, the strongest correlation was observed with the visceral fat (Q < 0.15) which is generally in accordance with the findings from the db-RDA analysis. Three pathways (DENOVOPURINE2-PWY, PWY-841, PWY0-162) already indicated in the dbRDA triplot in the weaker correlation cluster (Fig. 3H, green color) showed the strongest relationship with VF (Rho = 0.39, Rho = 0.39, Rho = 0.36, Q < 0.15, respectively). Full results of the univariate analysis are shown in Additional file 1: Figs. S4 and S5.

**Fig. 4:**
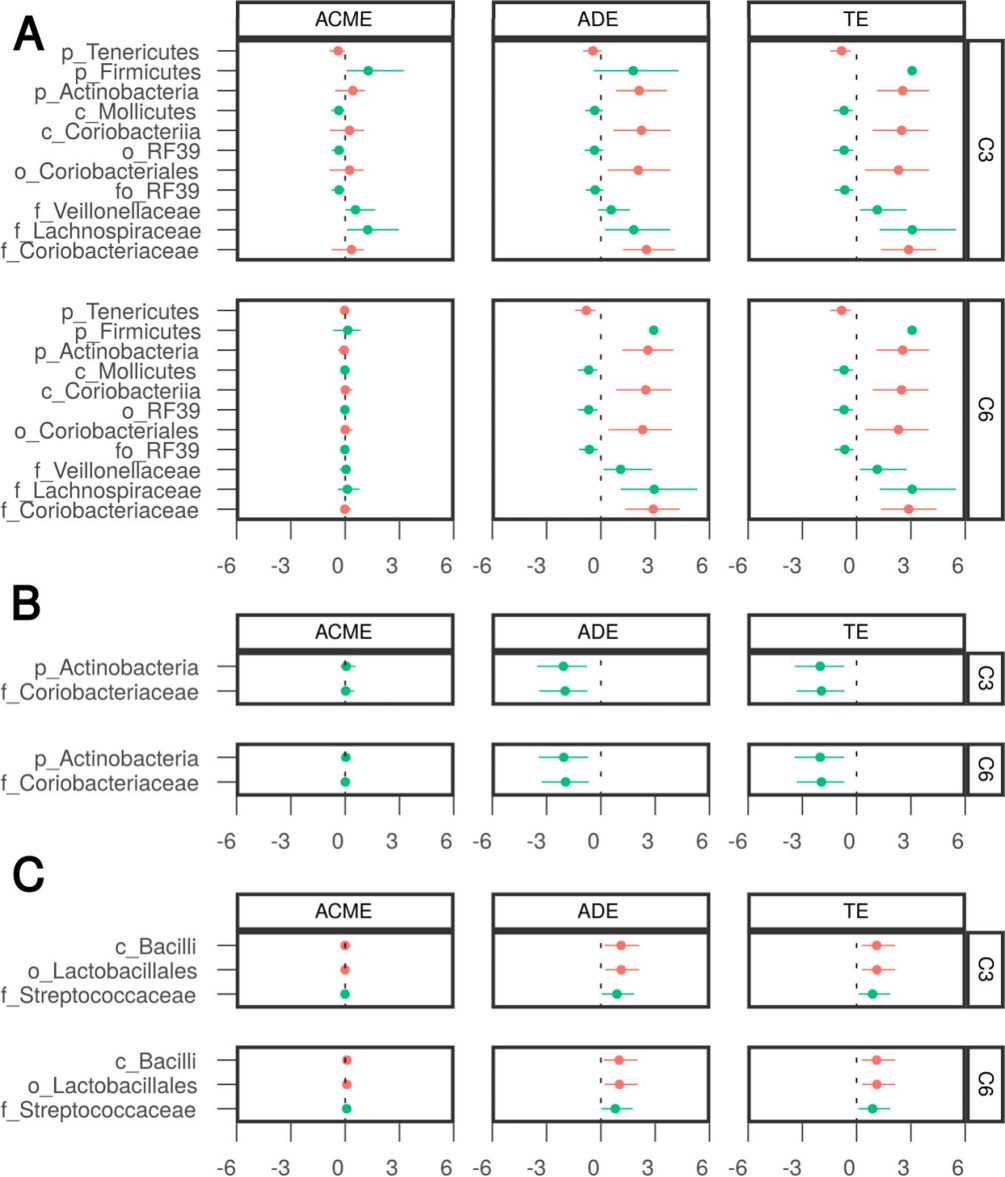
Mediation analysis for glucose, PWA PP and SBP—the effects and confidence intervals. **A** Glucose, **B** PWA PP, **C** SBP. Taxa correlating with glucose or PWA PP or SBP (FDR P < 0.1, Fig. 3C) are shown. Marked red taxa with FDR P < 0.05 (Fig. 3C). *ACME* average causal mediation effect, *ADE* average direct effect, *TE* total effect. Significance of each effect can be deduced from 95% confidence intervals not containing 0

Pairwise correlation analysis has indicated a relationship between the gut related features (taxa and pathways) and SCFA, especially C3 and C6 (Additional file 1: Figs. S4 and S5). As three parameters (glucose, PWA PP, SBP) were also associated with taxa (Fig. 3C), a mediation analysis was carried out to establish a statistical support for the causal effect of the gut microbes on parameters, in particular, whether the effect goes through the SCFA. Based on the results of pairwise correlation between bacterial features and parameters, as well as bacterial features and SCFA (Fig. 3C, Additional file 1: Figs. S4 and S5), three parameters (glucose, PWA PP and SBP) were selected as targets for the mediation analysis. The C3 and C6 were treated as potential mediators, whereas the gut microbes, exhibiting a correlation with these parameters, as independent variables. The main criterion for a selection of the taxa and parameters was maximizing a probability of the significant total effect (TE). As evidenced by the significant indirect (mediation) effects, the effect of family Lachnospiraceae (ACME 1.25, 95%CI (0.10, 2.97), phylum Firmicutes (ACME 1.28, 95%CI (0.23, 3.83)) and Tenericutes (ACME − 0.39, 95%CI (− 0.87, − 0.03)) on glucose was mediated by C3 (Fig. 4). The effect of Lachnospiraceae was incomplete (a significant direct effect as well, ADE 1.82, 95%CI (0.23, 3.83)), whereas the effects of Firmicutes and Tenericutes were fully mediated by C3 (an insignificant ADE). Statistically, there was no support for C6 transmitting the effect on glucose. Likewise, we could not establish a mediation role of the C3 or C6 on PWA PP and SBP.

## Discussion

This cross-sectional study revealed numerous correlations between the gut microbiota and risk factors for cardio-metabolic diseases. The microbiota of the surveyed women is characterized by high taxonomic diversity. We observed that alpha diversity was strongly and negatively correlated with LPS concentration and weakly correlated with other metabolic risk factors such as TG and visceral fat. Low bacterial richness was observed in metabolic disorders [4], inflammation [45] and obesity [28]. The gut microbiota plays an essential role in regulating the intestinal permeability of the intestinal mucosa, and a change in the microbiota community affects the mucosal barrier function of the gut [46, 47]. The elevated level of circulating insulin, typical for obesity phenotype, was found to increase the intestinal permeability, allowing bacterial toxins, such as LPS, to leak into the circulation, which, in turn, initiated a cascade of inflammatory reactions, thus, explaining the subclinical inflammation present in obese and insulin-resistant patients [48]. In our study, stool calprotectin was positively correlated with vascular stiffness, which may also indicate the role of increased intestinal permeability in the pathogenesis of cardiovascular diseases.

We observed a positive correlation between the phylum Actinobacteria and the lower taxonomic groups belonging to the class Coriobacteria with blood glucose levels. These observations confirm the previously described association of this group of bacteria with obesity and metabolic disorders [28, 49]. In contrast, the Tenericutes phylum was negatively correlated with blood glucose concentration in our study. On the contrary, in the study by Yan et al., this phylum occurred more frequently in obese rats but decreased after inducing diabetes and then increased largely after sitagliptin treatment as compared to the diabetic [50]. *Bacteroides caccae*, which is negatively correlated with waist circumference, is involved in the degradation of mucus, which helps reduce intestinal inflammation by decreasing bacterial epithelial adhesion [51]. Class Bacilli and order Lactobacillales were positively correlated with SBP. Adnan et al. observed a strong positive correlation between SBP and the lactate-producing genus *Lactobacillus* [52]. *Lactobacillus* can also synthesize neurotransmitters in the autonomic nervous system [53]. Furthermore, plasma lactate levels have been associated with increased blood pressure [54].

On the other hand, administration of *Lactobacillus plantarum* 299v [55] and *Lactobacillus casei* [56] can lower systolic blood pressure. However, strain-specific effects can differ from the effects of bacterial order. Proteobacteria abundance correlated with free fatty mass is associated with a reduction in mucus production and impairment of the gut barrier, and low-grade inflammation and is also associated with metabolic diseases such as obesity [57].

Brahe et al., in a study conducted on a similar group of women, have also observed a relationship between other groups of bacteria and cardiometabolic risk factors. However, the observed compounds are related to other taxonomic groups of bacteria [2]. It should be emphasized that the microbiota analysis carried out by Brahe et al. differed in the methodology of bacterial DNA sequencing, analytical pipeline and statistical methods used. Inconsistent microbiome research results make it difficult to understand the exact relationship between the human gut microbiome and the resulting disease [58]. This may be due to selection bias, geographic differences, unknown confounding factors, taxonomic differences, or the lack of standard sample collection, processing and analysis methods [59]. For this reason, it is necessary to both standardize the research protocol [60] as well as use open databases containing the results of microbiome studies in various populations [61].

To assess the universality of our observations, which is essential in microbiome research [62], we compared our data with those contained in the free and open Qiita database (https://qiita.ucsd.edu). We related the microbiota composition observed in our study to results obtained in a similar cohort of women in the American Gut Project (The Microsetta Initiative—Researching Global Microbiomes, https://microssetta.ucsd.edu), with both high and normal BMI. However, our results differed significantly at various taxonomic levels in both the high and normal BMI groups. Of particular note is the much lower percentage of Proteobacteria and the higher percentage of Actinobacteria observed in our study. Obesity is associated with various gut microbiota composition profiles [63]. These differences may have originated from the distinctness of the geographical locations of the investigated populations. The microbiome is not only distinct in Western and Eastern populations [64] but also differs between countries with similar lifestyles (e.g. USA and UK) and regions of the same country [42]. It should also be emphasized that we compared different variable regions of the 16S gene (V1- V2 vs V4), which may be a source of differences in the taxonomic composition of the bacteria [65]. This can introduce inaccuracies while assessing taxonomy [66] and further create problems when comparing the results of different studies, performing meta-analyses, and drawing generalized conclusions about the importance of microbiota in different diseases. Functional profile analysis seems more valuable than taxonomic analysis for this purpose [67]. The results are consistent with our previous observations in a Polish population [68] and in postmenopausal women [69].

However, in order to fully relate the information we obtained to a healthy population, a control group consisting of healthy and otherwise matched postmenopausal women of normal weight would be necessary.

An essential element of microbiota research is metabolomic analysis. In our study, we used both bioinformatics tools that can be utilized to assess the metabolic potential of the tested bacteria, as well as the SCFA analysis in the stool. We have shown that different metabolic pathways involved in gut microbiota can be correlated with cardiometabolic risk factors. These include pathways mainly related to lipid, bicarbonate, biotin and nucleotide metabolism. The strong positive correlation between biotin biosynthetic pathways and visceral fat may be of particular interest. This is in line with observations made by Wu et al. [70], who observed an association between the abundance of bacterial biotin biosynthesis pathways in individuals with different failures of glucose metabolism. Of note, intestinal bacteria produce biotin during insufficient supply [71]. However, this problem is not resolved, as Belda et al. [72] observed a reduced abundance of biotin-producing bacteria in severely obese individuals. In addition, they found reduced blood concentrations of this vitamin. Of note, Belda et al. used metagenomic analysis and employed counts of faecal microbial cell density. Our analysis was based on amplicons and determining the relative abundance of bacteria; moreover, women not only with severe obesity were enrolled in our study. These might stand for the discrepancies in these results. Despite the confirmation of biotin deficiency in T2D [73] and the beneficial effects of biotin supplementation on glucose metabolism [74–76], further research concerning the role of gut bacteria in biotin metabolism and its association with metabolic disorders and obesity is necessary to search for markers of these disorders and therapeutic possibilities. Also, purine metabolism was found to be positively associated with visceral fat. Altered purine metabolism was observed by Concepcion et al. [77] in a cohort of youths with T2D. Of note, hyperuricemia has been linked to visceral fat accumulation, and uric acid serves as the primary metabolite of balanced purine dietary uptake, synthesis, and excretion [78]. We also found that alpha diversity and abundance of numerous bacteria are correlated with faecal SCFA. Alpha diversity is negatively correlated with faecal propionic acid and positively with valeric and hexanoic acid. The correlations of other bacterial groups apply to those considered SCFA producers as well as to others. Interestingly, the abundance of some bacteria considered to be SCFA producers negatively correlates with the faecal SCFA. Similar situations occur in other populations, for example, in people with Parkinson's disease [79], which can be explained by the disturbed composition of bacteria in pathological states. Another factor that may influence the nature of the correlation between bacterial abundance and SCFA is a significant limitation of faecal SCFA analysis. The content of SCFA in the stool results from the production of these compounds by the intestinal bacteria, their absorption and their expenditure in situ in the gastrointestinal tract [80]. Faecal SCFA excretion results may not correspond to those measured in blood, which may better reflect SCFA production and absorption. Vogt and Wolever [81] showed that the rates of acetate absorption and excretion are inversely correlated. In studies that measured SCFA in the circulation compared to the faeces, slightly different conclusions were drawn about the relationship of SCFA with obesity and cardiometabolic health. Boets et al. found that obese people have lower plasma concentrations of propionate and butyrate than lean people [82]. Moreover, Layden et al. found that serum acetate concentration was inversely related to fasting and 2-h insulin levels and visceral adipose tissue [83]. It follows that the most reliable information can be obtained by analyzing the concentration of SCFA to provide an insight into all the processes of their transformation in the body. It can be assumed that pathological processes can lead to disorders of SCFA metabolism or absorption, which makes these compounds a potential marker of various disease states.

We have not confirmed a correlation between SCFA and cardiometabolic risk factors. SCFA are considered an important metabolite of gut microbiota with a beneficial effect on health. On the other hand, as a source of energy, they may be associated with the occurrence of overweight or obesity. The association of SCFA with metabolic disorders and obesity is, therefore, ambiguous. SCFA play regulatory functions in the lipids, cholesterol and glucose metabolism, immune response and gut barrier integrity. Other studies showed that SCFA in faeces were negatively correlated with adiposity parameters such as BMI, VAT and waist circumference [84, 85]. SCFA have been suggested to mediate the activation of G protein-coupled receptors, such as GPR41 and GPR43, inhibit fat accumulation in adipose tissue and accelerate the metabolism of unincorporated lipids as well as glucose in other tissues, leading to a subsequent improvement in insulin sensitivity [86, 87]. The release of gut-derived satiety hormones like glucagon-like peptide-1 and peptide YY have also been implicated in this action [88, 89]. SCFA also play a role in the balance of fatty acid synthesis, fatty acid oxidation, and lipolysis in the body's tissues through peroxisome proliferator-activated receptors [90]. Dietary supplementation with acetate, propionate, butyrate or their mixture can significantly inhibit the body weight gain induced by high-fat diet feeding [91].

On the other hand, the study performed in monozygotic twin pairs confirmed the positive effects of SCFA on obesity [92]. In other studies, higher levels of SCFA in the stool were found in overweight or obese subjects compared to lean subjects [26, 27, 93–95]. In addition, Turnbaugh et al., in an experimental study, showed a relationship between obesity, the gut microbiome and overproduction of SCFA [6]. However, the relationship between SCFA and metabolic disorders in postmenopausal women can be affected by decreased estrogen production, which causes intestinal dysbiosis [96]. The postmenopausal gut microbiota contains fewer SCFA-producing bacteria [69, 97–99]. It is therefore difficult to establish a clear relationship between faecal SCFA and cardiometabolic risk factors. To shed light on causal mechanisms, we performed a mediation analysis, which shown that the effect of Lachnospiraceae, Firmicutes and Tenericutes on plasma glucose may be translated (partially or fully) by propionate. However, these results should be interpreted with caution, given certain limitations. First, mediation analysis provides only statistical support for a possible underlying causal mechanism. Thus, carefully designed experimental studies need to be conducted to establish such relationships.

The main limitation of this study is the lack of a control group consisting of normal weight healthy and otherwise matched postmenopausal women; however, taking into account the multifactorial nature of the analysis, this task was not easy. Moreover, the study aimed to analyze the correlation in a given cohort and not the differences between the cohorts, especially since the problem of appropriate matching of healthy control was difficult to solve in such studies. The main reasons for the reduced sample size were the strict inclusion and exclusion criteria and limited resources. Furthermore, mechanistic studies using a germ-free mouse model are required to confirm the results. Finally, it would be beneficial to compare our results with a similar cohort [100]; however, such head-to-head studies have not been conducted. Other limitations include 16S rRNA sequencing of V1–V2 regions (in the Polish population, the most suitable region is not defined), which provides limited insight into microbiota function [101], and further metabolome and immunome analysis are required.

## Conclusions

We can conclude that microbiota composition and metabolic potential are associated with cardiometabolic risk factors and faecal SCFA concentration in obese postmenopausal women. However, there is no unequivocal relationship between faecal SCFA and the marker of intestinal barrier integrity and cardiometabolic risk factors. We also found that comparing results obtained in a study cohort with raw data contained in an extensive reference database was not informative regarding comparing stool microbiota between obese and non-obese patients, so appropriately matched control groups should be used in microbiota studies. Our study provides the translational significance. Future works might explain further whether the microbiota and its metabolic potential may be treated as markers of metabolic disorders in obese postmenopausal women. If these observations are confirmed, the creation of dysbiosis indices associated with the occurrence of these disorders could be used to develop appropriate dietary strategies and to monitor the health of women during this period of life. They may also serve as a basis for developing pre-, pro- and postbiotics that could reduce the risk of cardiovascular diseases.

## Supplementary Information

**Additional file 1: Figure S1.** Flow chart of the study population. **Figure S2.** Heatmaps of the rarefied abundance at different taxonomic levels. **Figure S3.** Alpha-diversity correlation with SCFAs. **Table S1.** db-RDA models summary. **Figure S4.** Correlations between bacterial taxa and anthropometric, physiological and biochemical parameters. **Figure S5.** Correlations between MetaCyc metabolic pathways and anthropometric, physiological and biochemical parameters. **Table S2.** Phylum relative abundance.

## Data Availability

The datasets presented in this study can be found in online repositories. The names of the repository and accession number can be found below: https://www.ebi.ac.uk/ena/browser/view/PRJEB48670.

## Abbreviations

CMRF: Cardiometabolic risk factors
CVD: Cardiovascular disease
T2D: Type-2 diabetes
SCFA: Short chain fatty acids
IBD: Inflammatory bowel diseases
ELISA: Enzyme-linked immunosorbent assay
OUT: Operational taxonomy unit
db-RDA: Distance-based redundancy analysis
DV: Dependent variables
IV: Independent variable
TE: Total effects
ACME: Average causal mediation effect
ADE: Average direct effect
FDR: False discovery rate
TM: Thrombomodulin
vWF: Von Willebrand factor
VEGF: Vascular endothelial growth factor
PWV: Pulse wave velocity
PWA AP: Pulse wave analysis augmentation pressure
PWA Alx: Pulse wave analysis augmentation index
PWA SP: Pulse wave analysis systolic pressure
PWA PP: Pulse wave analysis pulse pressure
LPS: Lipopolysaccharide
Il-6: Interleukin-6
TNF: Tumor necrosis factor alpha
CRP: C-reactive protein
DBP: Diastolic blood pressure
SBP: Systolic blood pressure
HR: Heart rate
TG: Triglycerides
HDL: High-density lipoprotein cholesterol
LDL: Low-density lipoprotein cholesterol
TC: Total cholesterol
FFMH: Fat-free mass hydration
FFM: Fat-free mass
FM: Fat mass
TBW: Total body water
WC: Waist circumference
BMI: Body mass index
