# Supplementary material for: Analysis of correlations between gut microbiota, stool short chain fatty acids, calprotectin and cardiometabolic risk factors in postmenopausal women with obesity: a cross-sectional study
Source: J Transl Med. 2022 Dec 12;20:585. doi: 10.1186/s12967-022-03801-0 (PMC9743526; doi:10.1186/s12967-022-03801-0)
Supplement: Supplementary file 1 — Additional file 1: Figure S1. Flow chart of the study population. Figure S2. Heatmaps of the rarefied abundance at different taxonomic levels. Figure S3. Alpha-diversity correlation with SCFAs. Table S1. db-RDA models summary. Figure S4. Correlations between bacterial taxa and anthropometric, physiological and biochemical parameters. Figure S5. Correlations between MetaCyc metabolic pathways and anthropometric, physiological and biochemical parameters. Table S2. Phylum relative abundance. [file 12967_2022_3801_MOESM1_ESM.pdf]

Supplementary data

Analysis of correlations between gut microbiota, stool short chain fatty acids, calprotectine, and anthropometric, physiological and biochemical parameters in postmenopausal women with obesity: A cross-sectional study

Igor Łoniewski, Monika Szulińska, Mariusz Kaczmarczyk, Konrad Podsiadło, Daniel Styburski, Karolina Skonieczna-Żydecka, Paweł Bogdański

Supplementary Figure 1. Flow chart of the study population

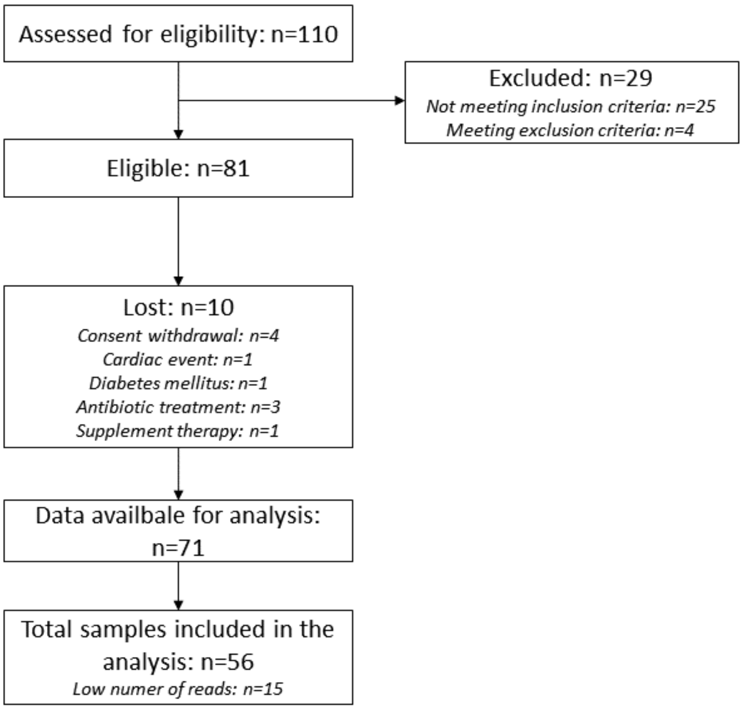

Supplementary Figure 2. Heatmaps of the rarefied abundance at different taxonomic levels

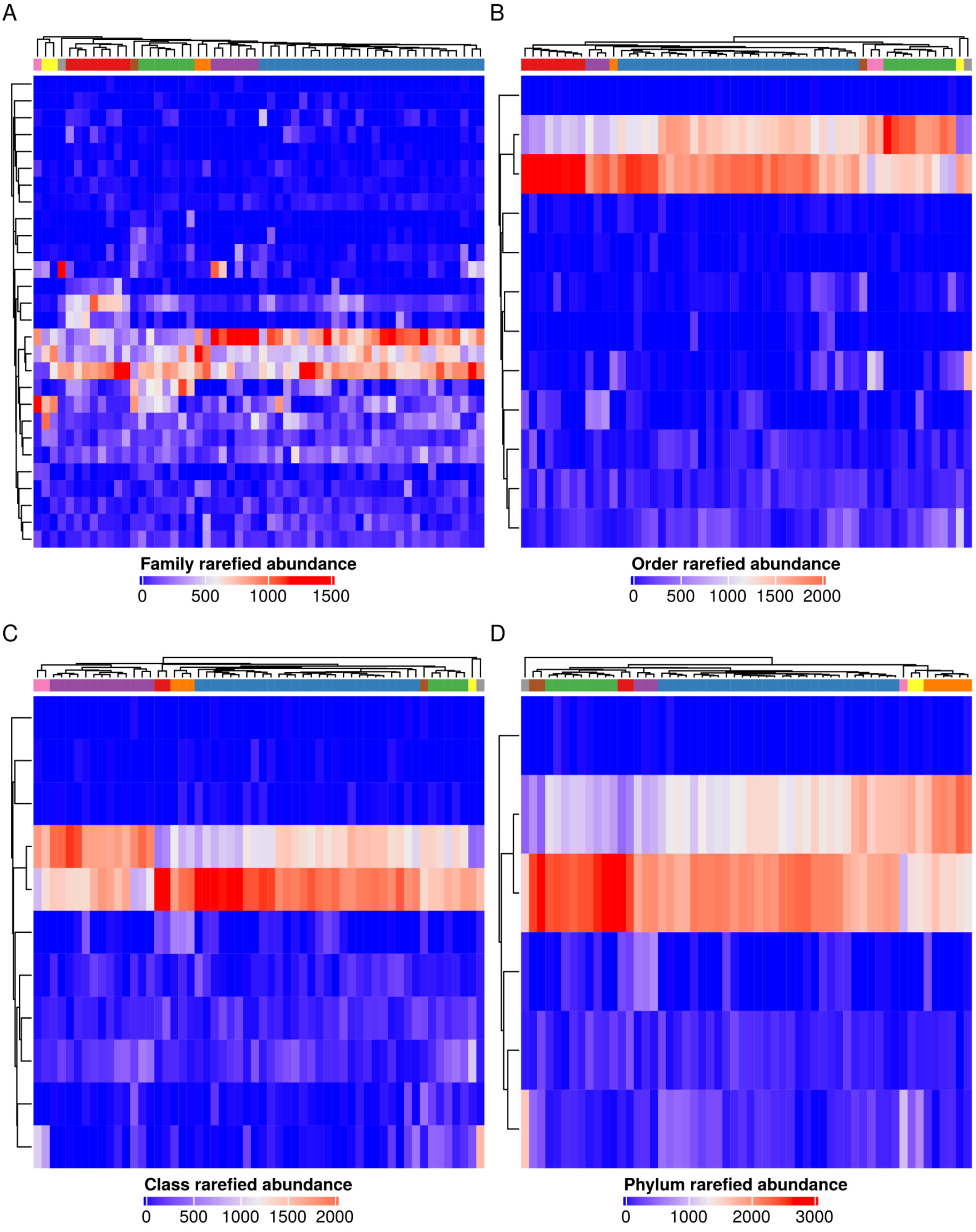

Columns (samples) and rows (taxa) were subjected to average linkage method and genus-level Bray-Curtis distance hierarchical clustering, top annotation - color bars reflect identified clusters

Supplementary Figure 3. Alpha-diversity correlation with SCFAs

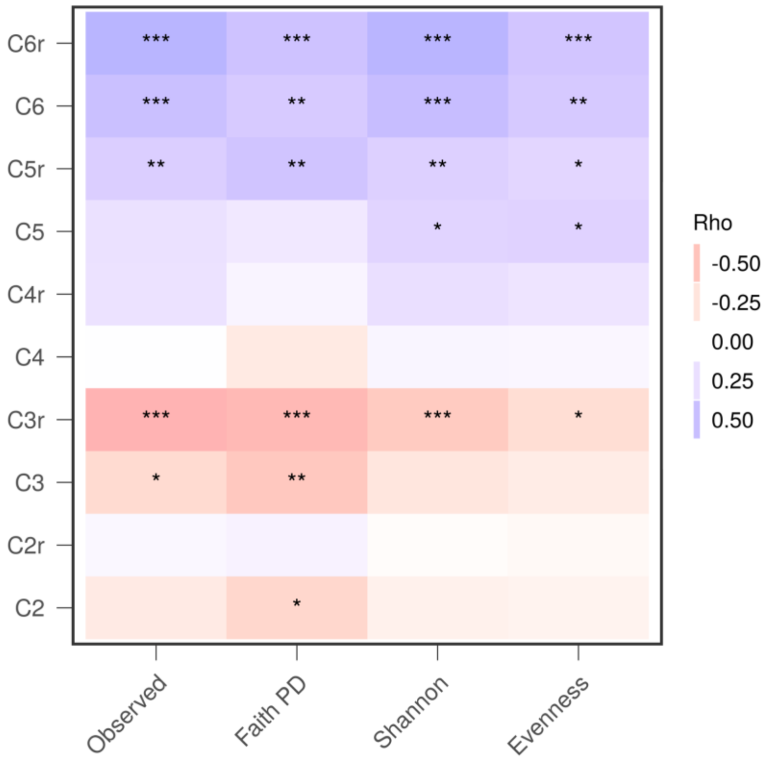

Mapping from p-value ranges to symbols: 0 - 0.001 '***', 0.001 - 0.01 '**', 0.01 - 0.05 '*', 0.05 - 0.1 '.', 0.1 - 1.0 No symbol; C2r - the ratio of acetic acid to all analyzed SCFAs, C3r - The ratio of propionic acid to all analyzed SCFAs, C4r - the ratio of butyric acid to all analyzed SCFAs, C5r - The ratio of valeric acid to all analyzed SCFAs, C6r - The ratio of hexanoic acid to all analyzed SCFAs.

Supplementary Table 1. db-RDA models summary

Model	Taxon	Variance explained - full model*	Simplified model - explanatory variables	Simplified model (R2)	Simplified model (adjusted R2)
Anthropometric	Genus	Constrained 0.06; Unconstrained 0.94 **P_{ANOVA}=0.202**	NULL	NULL	NULL
	Family	Constrained 0.07; Unconstrained 0.93 **P_{ANOVA}=0.189**	NULL	NULL	NULL
	Order	Constrained 0.05; Unconstrained 0.95 **P_{ANOVA}=0.522**	NULL	NULL	NULL
	Class	Constrained 0.05;Unconstrained 0.95 **P_{ANOVA}=0.510**	NULL	NULL	NULL
	Phylum	Constrained 0.06; Unconstrained 0.94 **P_{ANOVA}=0.368**	NULL	NULL	NULL
	MetaCyc	Constrained 0.12; Unconstrained 0.88 **P_{ANOVA}=0.003**	Visceral, WC	0.09381904	0.05962354
Physiological	Genus	Constrained 0.15; Unconstrained 0.85 **P_{ANOVA}=0.511**	NULL	NULL	NULL
	Family	Constrained 0.14; Unconstrained 0.86 **P_{ANOVA}=0.598**	NULL	NULL	NULL
	Order	Constrained 0.15; Unconstrained 0.85 **P_{ANOVA}=0.465**	NULL	NULL	NULL
	Class	Constrained 0.14;Unconstrained 0.86 **P_{ANOVA}=0.507**	NULL	NULL	NULL
	Phylum	Constrained 0.15 Unconstrained 0.85 **P_{ANOVA}=0.453**	PWA AP	0.06529508	0.04765914
	MetaCyc	Constrained 0.11; Unconstrained 0.89 **P_{ANOVA}=0.959**	NULL	NULL	NULL
Biochemical	Genus	Constrained 0.31; Unconstrained 0.69 **P_{ANOVA}=0.145**	Glucose	0.04262094	0.02308259
	Family	Constrained 0.32; Unconstrained 0.68 **P_{ANOVA}=0.112**	Glucose	0.06366385	0.04455495
	Order	Constrained 0.32; Unconstrained 0.68 **P_{ANOVA}=0.137**	NULL	NULL	NULL
	Class	Constrained 0.33; Unconstrained 0.67 **P_{ANOVA}=0.107**	NULL	NULL	NULL
	Phylum	Constrained 0.28; Unconstrained 0.72 **P_{ANOVA}=0.453**	NULL	NULL	NULL
	MetaCyc	Constrained 0.32; Unconstrained 0.68 **P_{ANOVA}=0.140**	Insulin	0.05522586	0.03594476

P_{ANOVA} Monte Carlo permutation test (number of permutations 999); variance inflation factor < 10 to avoid co-linearity issues: WC, subcutaneous, visceral from A group, all from the P group, all from the B group)

Supplementary Figure 4. Correlations between bacterial taxa and anthropometric, physiological and biochemical parameters

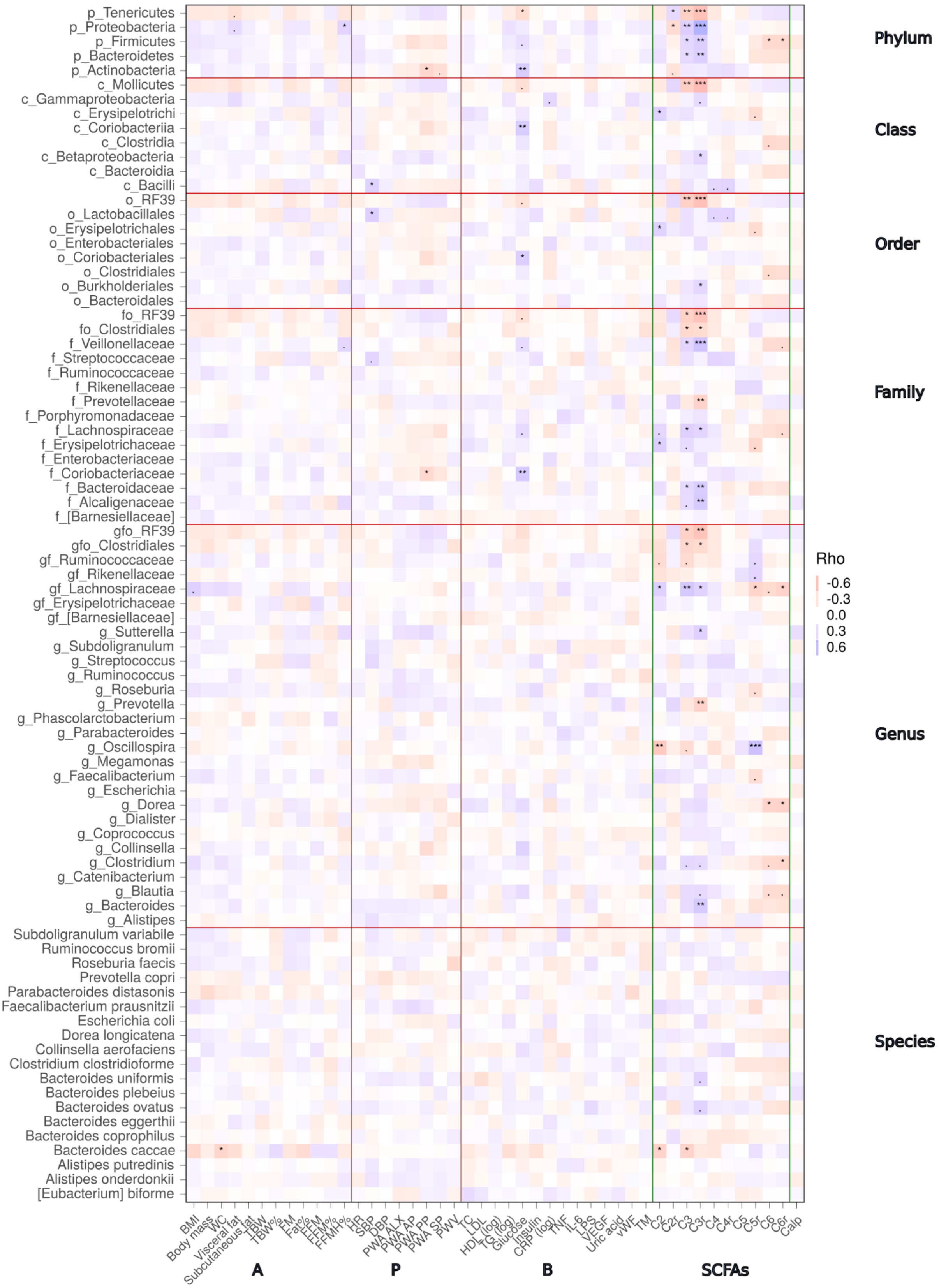

Spearman correlation of the bacterial abundance at the species, genus, family, order, class and phylum levels (separated by horizontal red lines) with anthropometric (A), physiological (P), biochemical (B) parameters (separated by vertical brown lines), short chain fatty acids (SCFAs) and calprotectin (separated by green line). Mapping from FDR adjusted P values ranges to symbols: 0 - 0.001 '***', 0.001 - 0.01 '**', 0.01 - 0.05 '*', 0.05 - 0.1 '.', 0.1 - 1.0 no symbol, Rho - Spearman correlation coefficient; C2r - the ratio of acetic acid to all analyzed SCFAs, C3r - The ratio of propionic acid to all analyzed SCFAs, C4r - the ratio of butyric acid to all analyzed SCFAs, C5r - The ratio of valeric acid to all analyzed SCFAs, C6r - The ratio of hexanoic acid to all analyzed SCFAs.

Supplementary Figure 5. Correlations between MetaCyc metabolic pathways and anthropometric, physiological and biochemical parameters

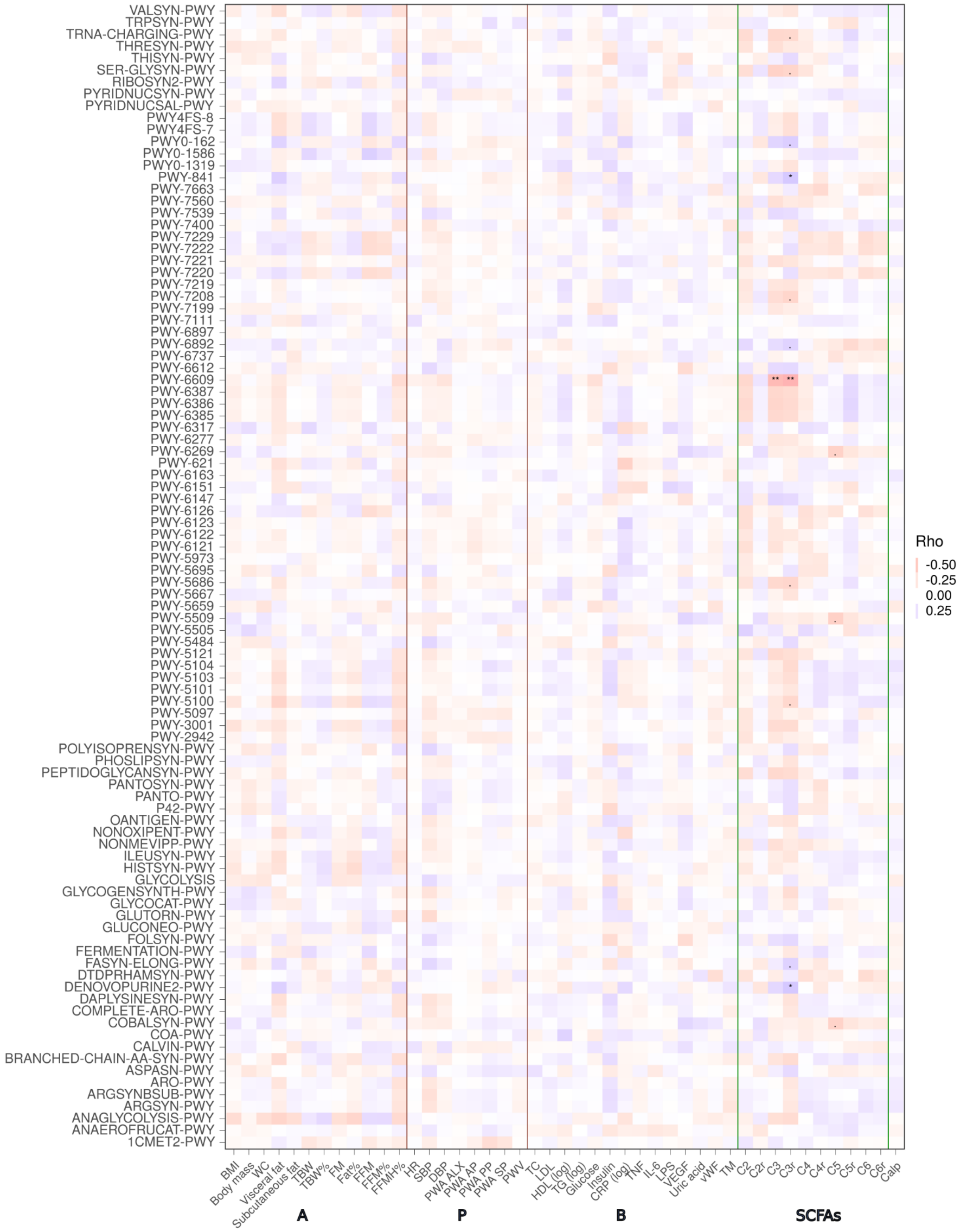

Spearman correlation of the PICRUSt2 predicted metabolic pathway abundance with anthropometric (A), physiological (P), biochemical (B) parameters (separated by vertical brown lines), short chain fatty acids (SCFAs) and calprotectin (separated by green line). Mapping from FDR adjusted P values ranges to symbols: 0 - 0.001 '***', 0.001 - 0.01 '**', 0.01 - 0.05 '*', 0.05 - 0.1 '.', 0.1 - 1.0 no symbol, Rho - Spearman correlation coefficient; C2r - the ratio of acetic acid to all analyzed SCFAs, C3r - The

ratio of propionic acid to all analyzed SCFAs, C4r - the ratio of butyric acid to all analyzed SCFAs, C5r - The ratio of valeric acid to all analyzed SCFAs, C6r - The ratio of hexanoic acid to all analyzed SCFAs.

Supplementary Table 2. Phylum relative abundance

	This study[1] (n=56)	BMI-NORMAL (n=1,293)	BMI-HIGH (n=875)
Actinobacteria	3.06%	1.09%	0.91%
Bacteroidetes	36.1%	27.8%	30.1%
Cyanobacteria	0.13%	0.14%	0.14%
Firmicutes	52.7%	34.2%	33.2%
Fusobacteria	0.016%	0.51%	0.23%
Proteobacteria	5.82%	32.8%	32.9%
Tenericutes	2.08%	0.80%	0.50%

BMI-NORMAL, BMI-HIGH > 0.1%; this study >0.1%, 1 - Time0

Class
This study vs BMI-HIGH: 997.4, p<0.0001
This study vs BMI-NORMAL: 691.4, p<0.0001
BMI-HIGH vs BMI-NORMAL: 203.0, p<0.0001

Order
This study vs BMI-HIGH: 994.3, p<0.0001
This study vs BMI-NORMAL: 647.8, p<0.001
BMI-HIGH vs BMI-NORMAL: -26.9, p=1

Family
This study vs BMI-HIGH: 2098.7, p<0.0001
This study vs BMI-NORMAL: 1509.3, p<0.0001
BMI-HIGH vs BMI-NORMAL: -222.9, p=1

Genus
This study vs BMI-HIGH: 2615.9, p<0.0001
This study vs BMI-NORMAL: 2031.9, p<0.0001
BMI-HIGH vs BMI-NORMAL: -1343, p<0.0001
